# Genome sequencing reveals variation of African swine fever virus in Nigerian outbreaks and identification of two major West African viral lineages

**DOI:** 10.1099/mgen.0.001636

**Published:** 2026-02-10

**Authors:** Ganih S. Joel, Anvou R. Jambol, Nenfort Daniel Jahota, Fengyi Li, Ruth N. Njuki, Lameck A. Odongo, Morenikeji Oluwatoyin Ruth, Gyang Godwill Ayuba, Daniel Geofrey ThankGod, Shuaibu Hafsat Jagab, Zheng-Fei Cai, Olufunke O. Oluwole, Bamidele Boladuro, M.O. Oladele-Buloka, Richard P. Bishop, Jianbo Li, Xinlou Yang, Adeyinka J. Adedeji, Pam D. Luka, Ya-Ping Zhang, Adeniyi C. Adeola, Min-Sheng Peng

**Affiliations:** 1 State Key Laboratory of Genetic Evolution & Animal Models and Yunnan Key Laboratory of Molecular Biology of Domestic Animals, Kunming Institute of Zoology, Chinese Academy of Sciences, Kunming, 650201, PR China; 2 Sino-Africa Joint Research Center, Chinese Academy of Sciences, Kunming 650204, PR China; 3 Kunming College of Life Science, University of Chinese Academy of Sciences, Kunming, PR China; 4 National Veterinary Research Institute, PMB 01, Vom, Nigeria; 5 State Key Laboratory of Genetic Evolution & Animal Models, Yunnan International Joint Laboratory of Zoonotic Viruses, Yunnan Key Laboratory of Biodiversity Information, Kunming Institute of Zoology, Chinese Academy of Sciences, Kunming 650201, PR China; 6 State Key Laboratory for Conservation and Utilization of Bio–Resources in Yunnan, School of Life Sciences, Yunnan University, Kunming 650091, PR China; 7 Institute of Agricultural Research and Training, Obafemi Awolowo University, Ibadan, Nigeria; 8 Washington State University, Pullman, WA, USA; 9 Bio-X Center for Interdisciplinary Innovation, Yunnan University, Kunming, PR China; 10 Department of Veterinary Physiology and Biochemistry, Faculty of Veterinary Medicine, Bayero University, Kano, Nigeria

**Keywords:** African swine fever virus, evolution, genotype I, genotype II, hybrid genome, Nigeria

## Abstract

African swine fever is a transboundary disease of wild boar and domestic pigs that is caused by the African swine fever virus (ASFV), with a mortality rate of 100 % in naïve animals. The ongoing spread poses a significant threat to food security and economic stability globally. In Nigeria, frequent outbreaks of the disease have been reported since 1997, and genetic analysis of Nigerian ASFV has defined virulent genotypes I and II. The disease has caused a significant impact in the pig-producing regions of the country, resulting in high economic losses. For improved control of the virus, a better knowledge of the virus’s genetic diversity is required. Here, we report five complete genome assemblies of ASFV from Nigeria, four genomes assembled with hybrid sequencing platforms and one using the Oxford Nanopore Technology, from different outbreaks between 2020 and 2024. Phylogenetic analysis indicates that these outbreak strains are of genotypes I and II, showing a striking sequence similarity to Benin 97/1 and Nigeria-RV502 of ≥99.90 %. Evolutionary analysis identified CAM1994 and Mauritius MAU/01/2007 to be the closest to the common ancestor of the circulating virus strains in Nigeria. The study reports for the first time the simultaneous occurrence of genotypes I and II in Nigeria. The study documents the evolutionary pattern of the two circulating genotypes in Nigeria and the West African region, allowing monitoring of the transmission dynamics, which will help strengthen surveillance and disease control.

**Impact Statement**

The study reports high-quality genomes of genotypes I and II in Nigeria, genotype I representing the first reported complete genome, confirming the co-circulation of the two genotypes in the country. The studies also help in tracing the transmission dynamics of both genotypes I and II across West African countries, including Nigeria, Cameroon, Benin and Ghana. These data fill a gap in the field of ASFV genomics by adding to the database of whole genomes of the virus from West Africa. Phylogenetic analysis of the data of both genotypes I and II revealed how the two virus genotypes spread within the continents of Africa, Europe and Asia. The study will significantly contribute to food security, ASFV outbreak responses and government control policies.

## Data Summary

The published complete genomic information of ASFV is listed in Table 1. We deposited the raw sequencing data to the Genome Sequence Archive of the National Genomic Data Center under the BioProject accession no. PRJCA041468. The five *de novo* genome sequence data are available in the National Center for Biotechnology Information with accession numbers PV842496/Nigeria-613, PV842497/Nigeria-DDS943, PV842498/Nigeria-LA74, PV842499/Nigeria-VPD805 and PV842500/Nigeria-DDS922.

**Table 1.** Assembly information of five Nigerian African swine fever virus genomes

Sample ID	Platform	Genome size	Contigs>800 bp	blast hit (strain)	% Identity	Indels per 100 kb	G+C %	ORFs	Assembler	Polishing tools
Nigeria-LA74	ONT+Illumina	181,403	1	Nigeria-RV502	99.96	4.43	38.64	174	SPAdes	Racon+Medaka+Pilon
Nigeria-VPD805	ONT+Illumina	182,284	1	Benin 97/1	99.94	12.66	38.58	153	SPAdes	Racon+Medaka+Pilon
Nigeria-DDS922	ONT	181,900	7	Nigeria-RV502	99.99	13.52	38.92	171	Flye	Flye+Racon+Medaka
Nigeria-DDS943	ONT+Illumina	182,033	1	Benin 97/1	99.90	13.18	38.57	152	SPAdes	Racon+Medaka+Pilon
Nigeria-613	ONT+Illumina	180,326	1	Nigeria-RV502	99.98	6.10	38.67	173	SPAdes	Racon+Medaka+Pilon

## Introduction

African swine fever (ASF) is a contagious transboundary disease responsible for significant economic losses in domestic pigs, and it is a notifiable disease of the World Organization for Animal Health. The disease is caused by African swine fever virus (ASFV), a contagious virus that poses a significant threat to the swine industry globally. The virus causes a haemorrhagic disease that has a devastating impact on naïve domestic pigs, with a mortality rate approaching 100 % [1]. Soft ticks (*Ornithodoros* species) and warthogs (*Phacochoerus africanus*) are known to be the primary hosts of the virus [2]. As the only member of the *Asfarviridae* family, ASFV is a large double-stranded DNA virus with a genome size of 170–194 kb, encoding over 150 proteins [3]. With a capsid structure resolved to be 4.1 angstroms, building from 17,280 proteins [4] Traditionally, genotyping has been performed using the 3′ end of the *B646L* gene that encodes the p72 capsid protein, which has so far identified at least 24 genotypes [1,5] However, recent whole genome re-analysis of genotype XVIII (NAM P1/1995) has shown that it is a mixture of genotypes I and VIII, which indicates that the number of distinct genotypes is 23 [6]. Diagnosis of the virus is based on the viral *EP402R* gene, which encodes *CD2v*, a haemadsorption-associated protein expressed on the surface of infected porcine erythrocytes [7,8]. The viral genome is divided into five major segments: the right variable region (RVR), central conserved region (CCR), left variable region (LVR) and two inverted terminal repeat regions (ITR) at 5′ and 3′ ends. RVR and LVR are the more diverse parts of the genome containing five major multi-gene families, which vary in copy number between isolates (*MGF*). CCR houses genes that function in viral replication and genome assembly [9]

Since the first report in 1921 from Kenya [10], ASFV has maintained circulation in domestic pigs in sub-Saharan Africa [11]. The virus has undergone rapid geographical spread, with the first wave outside Africa identified as P72 genotype I in 1957 in Lisbon (Portugal). The disease reappeared again, represented by the Lisbon 1960 isolate [12], is responsible for the widespread spread to many European countries, including Italy, France, Belgium, the Netherlands, Spain and also South America [13]. In the mid-90s, the disease had been eradicated in Europe and America, except Sardinia, where the virus remained endemic [14] until 2024, when it was eradicated (https://www.sanidadanimal.info/en/712-sardinia-free-asf). However, in June 2007, genotype II was reported in Georgia [15]. Since then, the virus has spread across the countries of Europe, including Russia. In 2018, ASFV was reported in China, the world’s largest pig-producing country, and has now spread to other neighbouring Asian countries [16,17]. By 2021, outbreaks were reported in the Caribbean countries of Haiti and the Dominican Republic [18,19]. In West Africa, genotype I was introduced in 1959 through Senegal, which belongs to serogroup 1 [20]. From 1996 to 2002, the virus spread again to other West African countries, including Côte d’Ivoire, Benin, Nigeria, Togo, Ghana and Burkina Faso, known to be serogroup 4 [21]. Recently, genotype II was reported in Nigeria [22]. Despite the considerable genetic diversity of the virus in Africa, only 17.5 % of the genomes (61 out of 348) available in the National Centre for Biotechnology Information (NCBI) database represent African strains. Only 7.47 % (26 out of 348) are from West Africa, with Benin 97/1 being the first reported whole-genome sequence [23]. This significant surveillance gap indicates a need for improved monitoring and data collection, including whole-genome sequences in the region.

In Nigeria, two near-complete genotype I sequences were reported in 2023 [24], and the first complete genotype II whole-genome sequence from Nigeria-RV5O2 was later reported in 2023. The genotype II strain shows unique characteristics when compared to Georgia 2007/1, with 6,535 bp deletions from position 11,760 to 18,295 and 688 bp deletions resulting in in-frame fusion of *MGF 360–3L* and *MGF 360–4L*, single-nucleotide insertion between *MG 360–1L* that changes to *KP360L*, a reverse complement duplication of *KP360L* of the 5′ end to the 3′ end *KP360R* and an in-frame fusion of *MGF 360–21R* and *MGF 360–2R* [25]. Genotype II has also been reported in Ghana and Benin [26]. The application of short-read data to assemble the ASFV whole genome can be challenging, due to background mammalian DNA, and cannot be used to determine inverted terminal repeat regions. Long-read data also present a limitation, in having a high error rate of 2–13 %. The combination of the two methods to generate a hybrid assembly provided powerful methods to comprehensively assemble a quality ASFV whole genome, allowing for accurate annotation and comparative analysis across different genotypes [27,28]. This method has been reported before in the Georgia 2007/1 strain and the Nigeria-RV502 strain assemblies [25,28].

Nigeria is the largest pig-keeping country in Africa [29], and ASFV is a major threat to pig production in the country. There is a significant gap in understanding the origin and the genetic diversity of the circulating genotypes in the country, and also, complete whole-genome sequences of genotype I from Nigeria have yet to be reported. Evolutionary analysis of genotype I, first reported by Adeola *et al.* [24], is currently limited to Italy, Portugal, Nigeria and Benin. A comparative evolutionary analysis that includes the circulating genotype II in West Africa has not been reported before. Lastly, a comprehensive comparative genomic analysis of the circulating strains over the years, particularly in Nigeria, has not been reported before.

Here we assemble high-quality hybrid genomes of genotypes I and II from Nigeria, leveraging the advantages of Illumina short-read sequencing and Oxford Nanopore long-read sequencing. We conducted the first analysis of molecular dating of genotype II from West Africa and expanded the analysis of genotype I to include Nigeria, Ghana, Benin and Cameroon. We also performed a comparative genomic analysis of the circulating genotypes from Nigeria. The findings of this study provide a broader understanding of the evolution and genomic diversity of ASFV in Sub-Saharan Africa.

## Methods

### Laboratory and computational workflow

All wet laboratory experiments, including DNA extraction and library preparation, were carried out in the Biotechnology Centre, National Veterinary Research Institute (NVRI), Vom, Plateau state, Nigeria, while all bioinformatic analyses (dry laboratory experiments) were conducted in Kunming Institute of Zoology, Chinese Academy of Sciences, Kunming, Yunnan province, China.

### Sample preparation

**Fig. 1.**
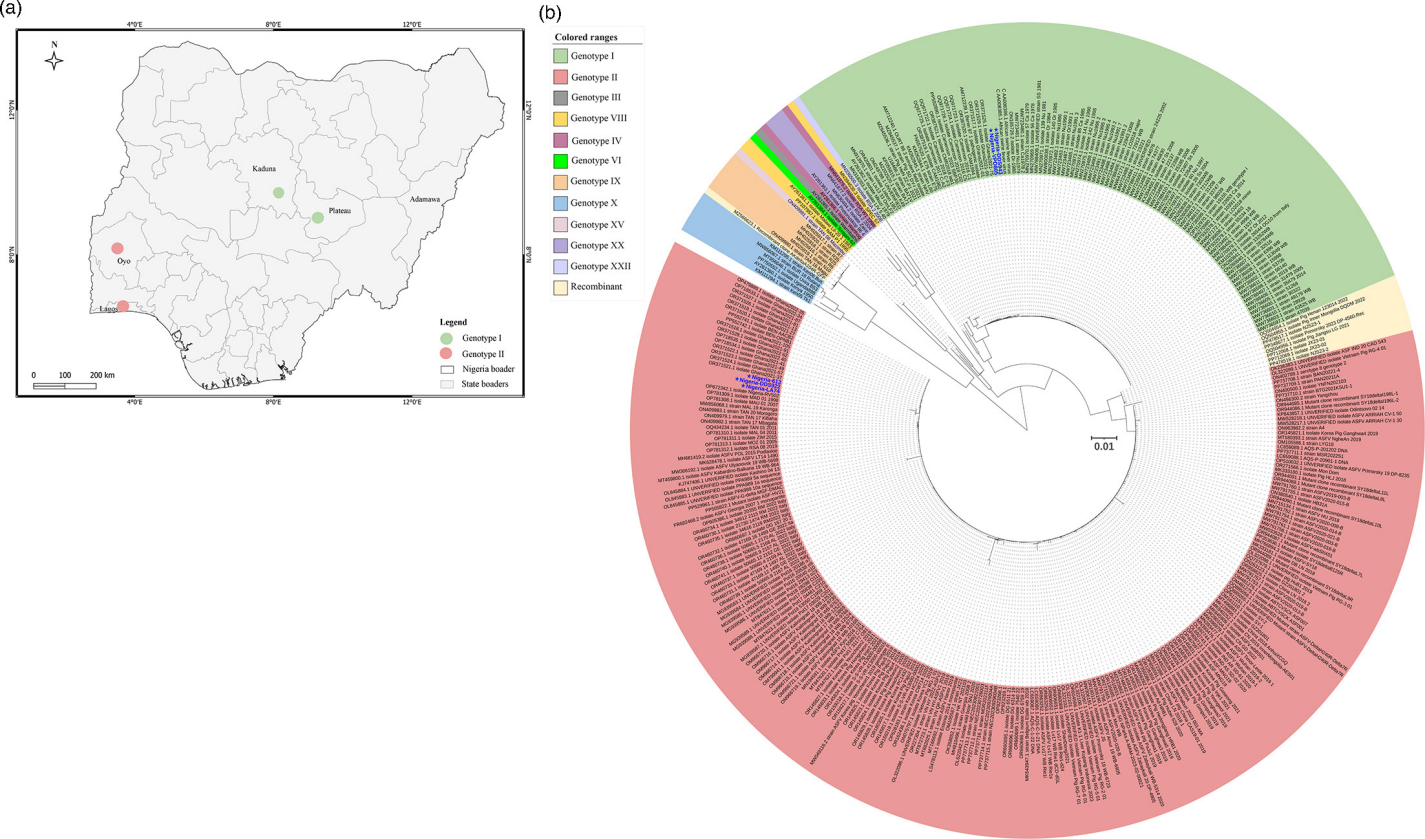
(**a**) Map of Nigeria showing the location of sample collection. (**b**) Maximum likelihood phylogenetic tree of the 5 new Nigerian ASFV genomes (highlighted in blue with an asterisk) together with 348 previously published ASFV genomes. The phylogenetic tree was constructed using the maximum likelihood method implemented in IQ-TREE2 and rooted on the midpoint. All branches shown are supported by 70 % or higher bootstrap values. Colours represent genotypes of ASFV based on the *p72* gene. Highlighted in light yellow are reported recombinant genomes. Seven recombinant genomes between genotypes I and II show as a distinct clade.

A total of five samples were collected from different locations in Nigeria from 2019 to 2024 (Fig. 1a). The samples are tissue (spleen, liver and mediastinal lymph nodes) and whole blood of infected pigs from different outbreaks.

### DNA extraction and PCR

DNA was extracted from the samples (blood and tissue) using the EasyPure Blood Genomic DNA Kit (TransGen Biotech), Cat. No. EE121. According to the manufacturer’s instructions, the genomic DNA was quantified using the Qubit 4 dsDNA Quantitation Kit from Thermo Fisher Scientific. The presence of ASFV was confirmed diagnostically with real-time PCR; the primer and the probe design were ASF-KING F 5′-CTGCTCATGGTATCAATCTTATCGA-3′, ASF-KING R, 5′-GATACCACAAGATCRGCCGT-3′, ASF KINGPB, 5′-(FAM) CCACGGGAGGAATACCAACCCAGTG-BH Q1-3′, designed by King *et al*., based on the *B646L (p72)* gene [30]. The PCR master mix is as follows: 2 μl of the template DNA was added to a total volume of 18 μl consisting of nuclease-free water (25 ml, New England Biolab), 10× Buffer, 25 mM MgCl2, 0.4 μM concentration of primers, 10 mM dNTPs (each) and 2.5 U μl^−1^ Taq polymerase (Thermo Fisher Scientific Walthan, MA, USA) in a total volume of 20 μl. The quantitative PCR was performed using the PikoReal 240 from Thermo Fisher Scientific, with cycling conditions as follows: initial denaturation at 95 °C for 5 min, followed by 40 cycles of 95 °C for 15 s and 58 °C for 30 s. Samples with a Cq value <25 were considered for library preparation and sequencing.

### Library preparation and sequencing

The Illumina library was prepared with the NEBNext UltraExpress^™^ DNA Library Prep Kit (NEB #E3325S/L). The sequencing was carried out by Illumina NovaSeq 600 (Novogen Singapore). The reagent kit and flow cell configuration were provided by the sequencing service provider and followed the manufacturer’s standard protocol for paired-end sequencing. Oxford Nanopore Technology library preparation and sequencing were performed using Ligation sequencing gDNA - NativeBarcoding Kit 96 V14 (SQK-NBD114.96 Oxford Nanopore Technologies, UK), sequenced with PromethION using R10.4 flow cell, Oxford Nanopore Technology platforms according to the manufacturer’s instructions on the DNA extract.

### Quality control and genome assembly

Raw reads from NovaSeq 600 Illumina were quality trimmed with Trimmomatic v0.36 [31], quality trimming -q 30 and a minimum read length of -l 30 to remove adaptors and low-quality bases;

.the data were inspected before and after trimming with FastQC v0.11.8. Nanopore raw reads fast5 were base-called with Dorado v0.9.0 (Oxford Nanopore Base Caller) (https://github.com/nanoporetech/dorado/); the Fastq files were processed with Porechop v0.2.4 with default parameters [32] and filtered with NanoFilt v1.42.0 with average reads’ quality score of -q 10 and minimum read length of -l 500 bp; and the data were also inspected before and after trimming with NanoPlot v1.42.0 [33]

To remove the host reads, Illumina and Oxford Nanopore trimmed data were mapped against ASFV genotypes I and II. Benin 97/1 and BA71 (genotype I) and ASFV Georgia 2007/1 and Nigeria-RV502 (OP672342) (genotype II) were mapped using BWA-MEM2 v2.2.1 [34] and Minimap2 v2.24 [35]. Samtools v1.9 parameter F −4 was used to remove the unmapped reads. Hybrid genome *de novo* assembly was performed using SPAdes v3.14.1 [36]. Note that Nigeria-DSS922 has only Oxford Nanopore raw data; it was *de novo* assembled with Flye v2.9.1 [37].

### Assembly mapping, error correction and determination of genome completeness

To enhance the accuracy of the assembled genome, a multi-step polishing approach was applied using both Oxford Nanopore and Illumina sequencing data. Oxford Nanopore data were aligned to the scaffold FASTA using Minimap2 v2.24. The initial polishing was performed using Racon v1.5.0, running three iterations with default parameters [38]. To refine the consensus sequence, the sequence generated from Racon was further polished using Medaka v1.11.3 (https://github.com/nanoporetech/medaka), three iterations, leveraging Nanopore data to improve base accuracy and quality of the assembly. Trimmed Illumina data were mapped against the Medaka polished FASTA file. The resulting align file was sorted and indexed with Samtools v1.9 with sort and index functions, Pilon v1.24 was used with default settings using the --fix all function. Three rounds of error correction were performed, using the indexed BAM alignment file [39]. Viral genome confirmation was determined using VirSorter2 v1.0.1 [40], and the genome completeness of the ASFV genome assembly was determined using CheckV v0.01 [41]. Genome completeness and genome orientation were further determined using Quast v5.2.0 [42]. All the confirmations were carried out with default parameters. Genome annotation was carried out using the Genome Annotation Transfer Utility GATU [43], adopting Nigeria-RV502 for genotype II and Benin 97 for genotype I, as references. All the annotation was manually checked and compared, genotype I with CAM1994 and genotype II with Georgia 2007.

### Genome alignment and phylogenetic analysis

A total of 348 complete and near-complete sequences were downloaded from the NCBI Nucleotide Database, including both field isolates and experimentally derived reference genomes under the taxonomy ‘taxid10497’ (https://www.ncbi.nlm.nih.gov/datasets/genome/?taxon=10497) accessed on 09 April 2025, as well as GeneBase (https://ngdc.cncb.ac.cn/genbase/search/gb/) for C_AA008386.1 and C_A008386.1, two near-complete genomes of genotype I from Nigeria accessed on 09 April 2025 (Table S1, available in the online version of this article). The sequences were compared against the five newly assembled whole genomes. Multiple sequence alignment was performed with MAFFT v7.5.11 online version (https://mafft.cbrc.jp/alignment/server/) accessed on 09 April 2025 [44]. This was followed by trimming the ambiguously aligned region of the multiple sequence alignment with trimAl v1.4.1 with the -automated1 function [45]. The aligned sequences were manually checked using Aliview [46].

A phylogenetic tree was constructed using maximum likelihood. The best-fit substitution model was determined using ModelFinder based on the Bayesian information criterion (BIC). Maximum likelihood was performed using IQ-TREE v2.2.0.3 with the best-fit model TVM+F+R5 chosen according to BIC, with 1,000 bootstrap support values [47]. The weakly supported branches (with a bootstrap value of less than 70%) were collapsed using the Biotree utility in BpWrapper [48]. The tree was rooted at the midpoint and visualized using the Interactive Tree of Life (ITOL) v6 [49].

### Molecular dating

A total of 35 whole genomes for genotype I and 39 for genotype II were determined using the Bayesian inference with the Markov Chain Monte Carlo (MCMC) method in BEAST v2.77 by constructing a divergence time tree. The data for genotype I was analysed with the GTR+G+I substitution model using the relaxed exponential clock with the coalescent Bayesian skyline model with an MCMC of 50 million. Genotype II was analysed with the GTR+G substitution model, relaxed clock log normal, coalescent constant population as prior, with MCMC 300 million [50]. An effective sample size >200 was used to assess the results, with 10 % burn-in using Tracer v1.7.2 [51]. The molecular dating tree was visualized with FigTree v1.4.4. (https://tree.bio.ed.ac.uk/software/Figtree/).

### Selection pressure

We compared two pairs of genomes from the most recently collected viral isolates to test for signatures of adaptive mutations. These pairs include Nigeria-VPD805 and Nigeria-DDS943 for genotype I and Nigeria-LA74 and Nigeria-613 for genotype II. For each pair, we identified nonsynonymous mutations on the most recent lineage by comparing with a closely related outgroup genome, including Benin 97/1 for genotype I and Nigeria-RV502 for genotype II. Genes that do not show 100 % identity between the two sister genomes were considered as candidate genes for selection pressure analysis. A total of eight genes (*DP71L, P1192R*, *B407L*, *MGF 505–2R*, *MGF 360–8L*, *B438L*, *I267L* and *E301R*) were extracted and re-aligned individually using a site model to identify key target amino acids under selection, and codon alignments were obtained using MAFFT. Selection pressure was determined using Datamonkey (https://www.datamonkey.org/) with the following methods: (i) mixed effects model of evolution (MEME), (ii) single likelihood ancestor counting (SLAC) [52] and (iii) fast unconstrained Bayesian approximation (FUBAR) [53]. Sites identified by one method were considered to be under diversifying positive selection.

## Results

### Sample, parameters and clinical presentation

A total of five samples were collected in Nigeria during the years 2020 to 2024 from three geopolitical regions, North Central, North West and Southwest (Fig. 1a). (i) LA74 was collected from the Southwest (Lagos state) during an outbreak in 2020 in Oke-Aro farm, from a herd of 155, of which 109 died, exhibiting symptoms of reddening of skin around the ear and eye, abortion, anorexia, loss of appetite, loss of weight and body weakness. (ii) VPD805 was collected from North Central (Plateau state) during a 2021 outbreak on a local pig farm. The observed clinical signs were reddening of the skin around the ear and eye, abortion, anorexia, loss of appetite and body weakness. (iii) DDS922 was collected during a 2022 outbreak from Southwest, Oyo state, in a farm with a herd size of 95, of which 73 died; the clinical signs observed are reddening of the skin around the ear and eye, abortion, anorexia, loss of appetite and vomiting. (iv) DDS943 was collected from North West, Kaduna state, during a 2023 outbreak from a farm with herd size 66, 20 affected and 3 died; the observed clinical signs were reddening of skin, abortion and anorexia. (v) 613 was obtained during a 2024 outbreak in Oke-Aro farm in Southwest, Lagos state. The observed clinical signs were reddening of skin around the ear and eye, abortion, anorexia, loss of appetite, loss of weight and body weakness. The sample was collected immediately after clinical signs were observed (Table 2).

**Table 2.** Epidemiological information of five Nigerian samples

NVRI lab. no.	Collection year	Sample type	Host	Sampling location	History	Real-time PCR (Ct)
**LA74**	2020	Spleen, liver and lymph nodes	Pig	Oke-Aro, Lagos state	Head size=155, affected=na, 109=died,	24.43
**VPD805**	2021	Liver, lungs and kidneys	Pig	Jos, Plateau state	Heads size=na, affected=na,	22.51
**DDS922**	2022	Spleen, liver and lymph nodes	Pig	Oyo state	Head size 95, affected=na, 73=died	22.34
**DDS943**	2023	Spleen, liver and lymph nodes	Pig	Kaduna state	Heads size=66, affected=20, 3=died	23.75
**613**	2024	Whole blood	Pig	Oke-Aro, Lagos state	Heads size=na, affected=na	20.63

### Whole-genome sequencing, hybrid genome assembly and annotation

To generate a high-quality assembled genome, two different technologies were employed: next-generation sequencing (NGS) using Illumina NovaSeq 600 and Oxford Nanopore Technology (ONT), UK. Nigeria-LA74: ONT generated raw data of 9.26 GB, which generated a total of 4,900,917 reads and 3,188 specific ASFV reads, while NGS generated raw data of 18.4 GB, which comprises a total of 280,603,556 reads with 375,188 reads specific to ASFV. Nigeria-VPD805: ONT total raw data 7.55 GB, which produced a total of 3,688,850 reads and 2,759 reads specific to ASFV and NGS with raw data 18.8 GB, having a total of 303,796,865 reads and 240,485 ASFV-specific reads. Nigeria-DDS922: total ONT raw data 8.14 GB with total reads 5,505,175 and 4000 reads specific to ASFV. Nigeria-DDS943: with ONT total raw data 11.0 GB, with total reads 6,801,349 and 4,933 reads specific to ASFV, while NGS contained raw data of 14.4 GB with total reads of 228,451,931, having 315,919 reads specific to ASFV. Nigeria-613: ONT with raw data 0.678 GB and total reads of 233,381 and 1,239 specific to ASFV, while NGS raw data amounted to 18.3 GB, comprising total reads of 290,862,169, with 456,677 reads specific to ASFV.

The ASFV-specific reads were used for hybrid *de novo* genome assembly. To correct sequencing errors by Nanopore, gaps, mis-assembly and misalignment from the draft hybrid assembled contigs were mapped back to the trimmed Nanopore raw reads, with further polishing with the trimmed Illumina raw reads. Nigeria-LA74 generated a single contig of 181,403 bp, 38.64 mol % G+C content, and showed 99.96 % sequence identity to Nigeria-RV502 and 99.87 % identity to Georgia 2007/1 according to blastn analysis of data in NCBI. Nigeria-VPD805 generated a single contig 182,284 bp, 38.58 mol% G+C content. When the sequence was searched against NCBI, it showed 99.94 % sequence identity to Benin 97/1, which is in genotype 1. Nigeria-DDS922 generated 7 contigs with a scaffold size of 181,900, 38.92 mol% G+C content. When blastn in NCBI, it showed 99.99 % identity to Nigeria-RV502 and 99.79 % similarity to Georgia 2007/1. Nigeria-DDS943 generated a single contig 182,033 bp, 38.57 mol% G+C content. When the sequence was searched against NCBI using blastn, it showed 99.90 % sequence identity to Benin 97/1. Nigeria-613 generated a single contig of 180,326 bp, 38.67 mol% G+C content. After blastn against NCBI, it showed 99.98 % sequence identity to Nigeria-RV502 and 99.94 % similarity to Georgia 2007/1. Note: Nigeria-DDS922 have been assembled using ONT only (Table 1).

### Phylogenetic analysis

The whole-genome phylogenetic analysis revealed that the five newly assembled genomes, Nigeria-VPD805 and Nigeria-DDS943, clustered in genotype I, while Nigeria-LA74, Nigeria-DDS922 and Nigeria-613 clustered in genotype II. The analysis grouped Nigeria-VPD805 and Nigeria-DDS943 as sister groups with the previously reported nearly complete genotype I from Nigeria C_AA008386 and C_A008386 collected in 2016; the whole genomes reported from Nigeria share a sister clade with that of other West African countries, Ghana2021-01, Benin 97/1, CAM1994, Ghana2021-75 and Ghana2021-95. The analysis also grouped Nigeria-LA74, Nigeria-DDS922 and Nigeria-613 as sister groups with the previously reported genotype II from Nigeria, Nigeria-RV502 and other West African genotype II from Ghana and Benin. The West African genotype II shares the same clade with that of Mauritius MAU/01/2007 and Madagascar MAD/01/1998, sharing a sister clade with that of Tanzania TAN/20/Morogoro and Malawi MAL/19/Karonga, suggesting Southeastern Africa is to be the possible origin of the recently reported genotype II from West Africa (Fig. 1b). The virus name and the corresponding country where the isolate was collected are clearly labelled in Table S1.

### Molecular dating

**Fig. 2.**
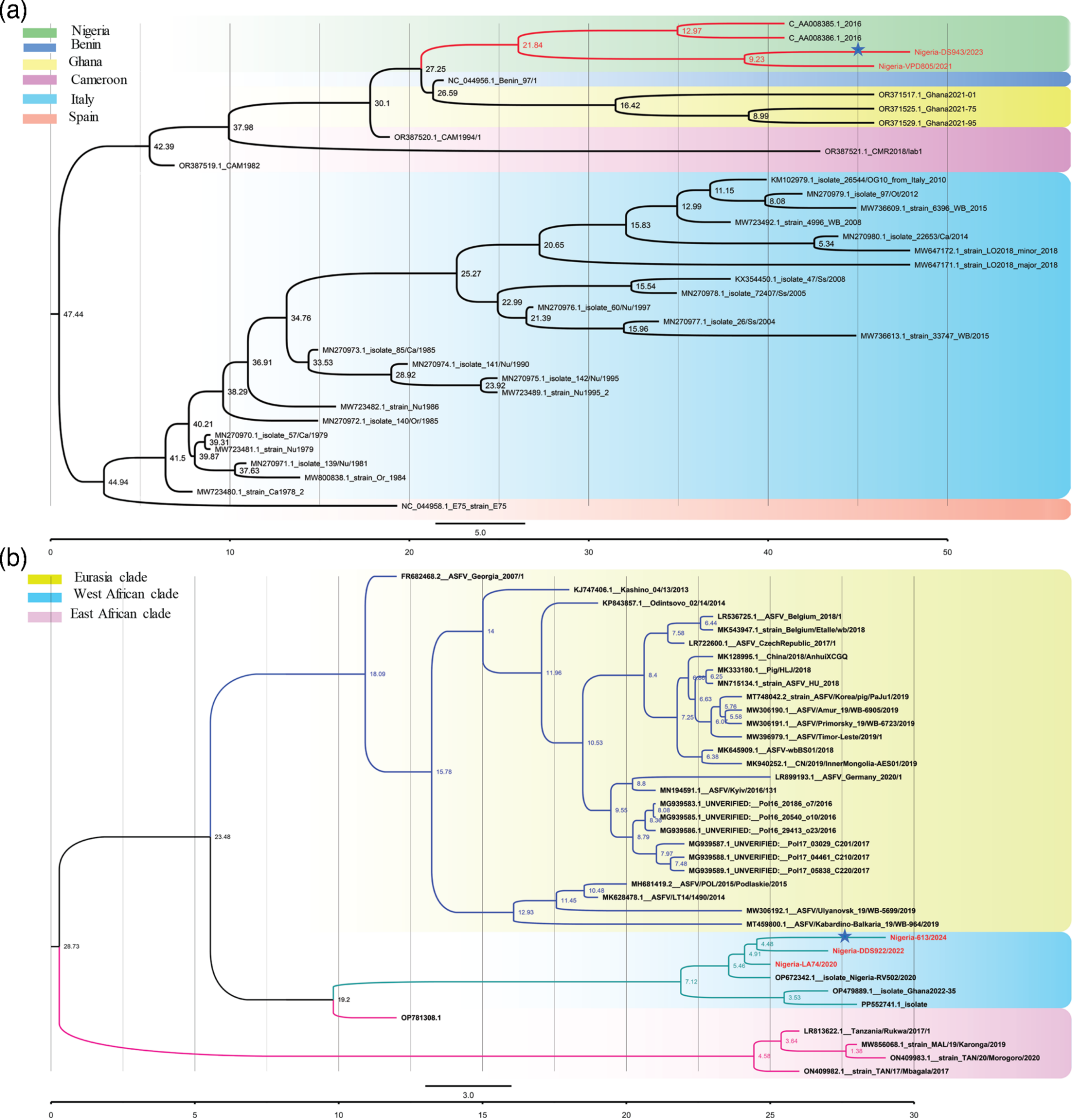
Timed phylogenies estimated using BEAST indicating divergence times among (**a**) genotype I isolates and (**b**) genotype II isolates. Five new genomes are highlighted in red. Two branches are marked with blue asterisks, indicating *n*=13 novel mutations associated with Nigeria-DDS943, the 2023 isolates, and *n*=14 novel mutations associated with Nigeria-613, the 2024 isolate (Tables S2 and S3). These latest mutational changes were identified according to the parsimony principle by comparing with their respective sister-group isolates (Nigeria-VPD805 for Nigeria-DDS943 and Nigeria-LA74 for Nigeria-613) as well as outgroup isolates (Benin 97/1 for Nigeria-DDS943 and Nigeria-RV502 for Nigeria-613).

To gain insight into the origin of ASFV in West Africa, we use the time of the most common ancestry (TMCA) to date back and reveal the ancestral lineage of the circulating strains of genotypes I and II. The genotype I strain in West Africa split from the European strain E75 (Spain), believed to have ultimately originated from Angola via Portugal, around 50 years ago. Further divergence within West Africa suggested that the pathogenic strain Benin 97/1 and Nigeria strains diverged from the CAM1994 strain around 30 years ago; this is close to the time of the second wave of the disease observed in West Africa in 1996 [54]. Nigeria and Benin strains diverged 27.25 years ago; this was around the reported outbreak of 1997 in Nigeria [55]. With further divergence within Nigeria, viruses emerged ~20 years ago. The mean evolutionary rate of genotype I was estimated to be 6.265×10⁻⁶ substitutions/site/year (95 % Highest Posterior Density interval (HPD): 5.0366×10⁻⁶ to 7.4912×10⁻⁶), consistent with the estimate reported by Adeola *et al*. at 6.3×10⁻⁶ substitutions/site/year (95 % HPD: 4.18×10⁻⁶ to 8.45×10⁻⁶) [24] Fig. 2a. In contrast, genotype II TMCA was estimated, revealing the divergence of African strain to that of Eurasian to 23.8 years ago, while within Africa, strain MAU/01/2007 (OP781308) from Mauritius, Southeastern Africa, diverged 19.17 years ago from the West African strain, with further divergence in West Africa between the strains of Nigeria and Ghana around 7.82 years ago, close to the report of the first outbreak of genotype II in Nigeria around 2019/2020. The mean evolutionary rate of genotype II was estimated to be 3.779×10^–5^ substitutions/site/year (95 % HPD: 2.0994×10^−5^ to 5.4091×10^−5^), suggesting a faster evolutionary dynamic than the report by Zhang *et al.* [56] at 1.312×10^–5^ substitutions/site/year (95 % HPD: 7.7432×10^–6^; 1.8733×10^–5^) [56] Fig. 2b. The evolutionary analysis results for genotypes I and II coincide with the previous outbreaks, suggesting that the accelerated evolutionary divergence observed in the genotype II strain may have been driven by an increased mutation rate as a result of exposure to a novel environment [57]. AFSV’s whole genome from Africa is underrepresented because of the limited number of whole genomes.

### Genetic variations

**Fig. 3.**
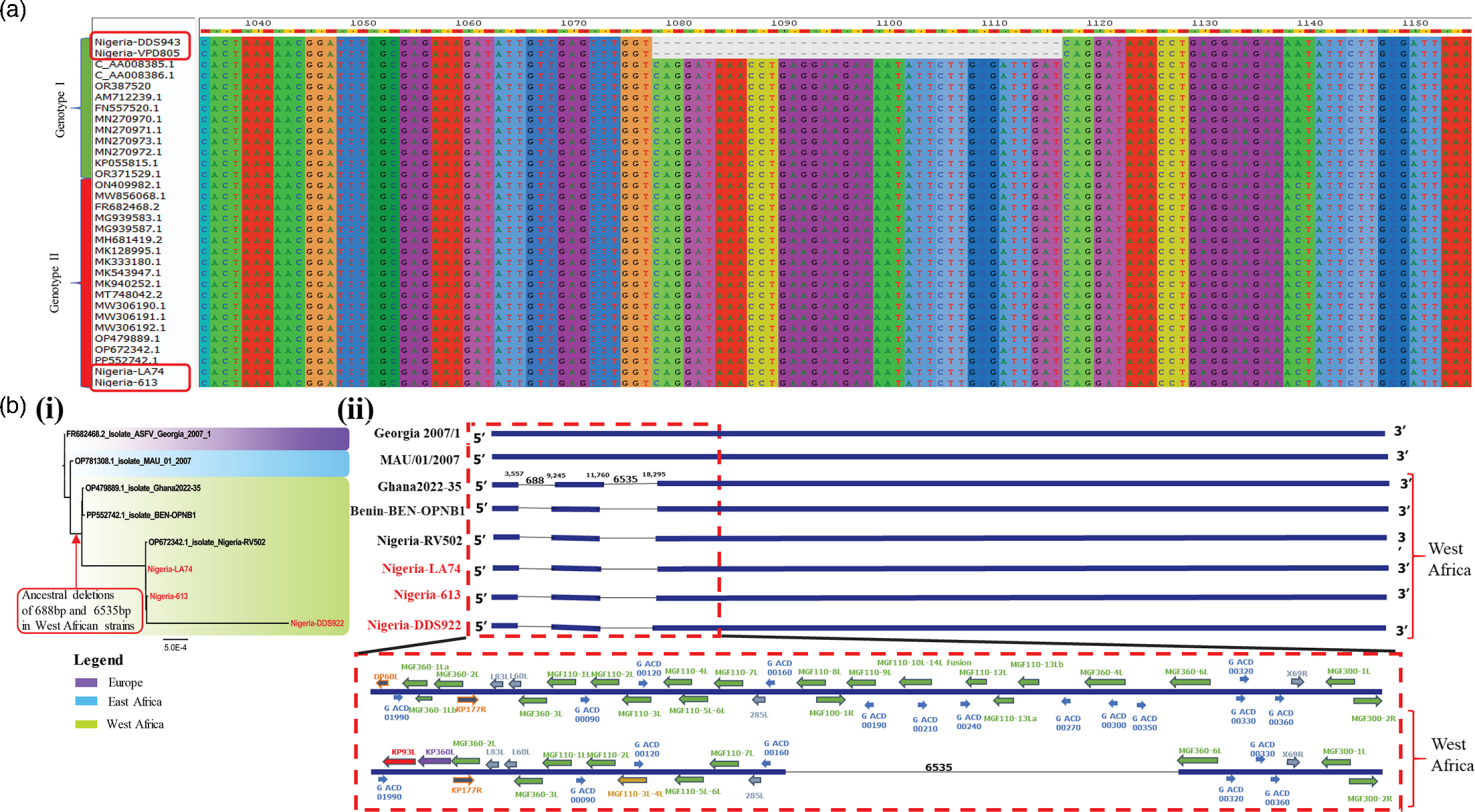
(**a**) Both genotype I genomes show a 38-nt deletion in gene *B475L* from position 1,077 to 1,115. (**b**) (**i**) Both genotype II genomes show genomic deletions of 688 bp and 6,535 bp. (ii) Phylogenetic analysis with closely related genomes suggests that these two large deletions occurred in the common ancestor of the West African lineage.

Genetic variation was identified in both genotypes I and II. Sequence comparison revealed that Nigeria-VPD805 has 36 SNPs and Nigeria-DDS943 has 39 SNPs when compared to Benin 97/1. Further comparison between the 2023 outbreak (Nigeria-DDS943) and the 2021 outbreak (Nigeria-VPD805) showed 13 novel SNPs on *MGF 360–1L*, *MGF 300–4L*, *MGF 505–4R*, *A151R*, *M448R*, *C962R*, *B407L*, *CP530R* and *DP71L* genes and 3 found in non-coding regions, as shown in Table S2. A 38-nt deletion was observed in the *B475L* gene in both the Nigeria-VPD805 and Nigeria-DDS943 (2021 and 2023) outbreaks of genotype I, from position 1077 to 1115, when compared with the previously reported genomes Fig. 3a. Genome comparison between Nigeria-RV502 and the newly assembled genome revealed 4 SNPs between Nigeria-RV502 and Nigeria-LA74, and 16 SNPs between Nigeria-RV502 and Nigeria-613. The genetic difference between the early outbreak of 2020, Nigeria-LA74, and the recent 2024 outbreak, Nigeria-613, showed that Nigeria-613 has unique SNP changes on *MGF 360–2L*, *MGF 110-10-14L* fusion protein, *MGF 110-13La*, *MGF 360–8L*, *MGF 360–13L*, *E183L*, *E301R*, *MGF 360–21R* genes and two from the non-coding region, *MGF 360–8L*, *E183L*, *E301R* and *MGF 360–21R* as shown in the Table S3.

Further genomic comparison of the newly assembled genotype II strains from Nigeria to the standard reference genome Georgia 2007/1 reveals a 6,535-nt deletion between positions 11,760 and 18,295. The genomic deletion resulted in the loss of 14 genes that include *MGF 110–8L*, *MGF 100–1R*, *MGF 110–9L*, *G ACD 00190*, *MGF 110–10* L-14L fusion protein, *G ACD 00210*, *MGF 110–12L*, *G ACD 00240*, *MGF 110-13La*, *MGF 110-13Lb*, *G ACD 00270*, *MGF 360–4L*, *G ACD 00300* and *G ACD 00350*. These genomic deletions are consistent across all the genotype II strains reported from West Africa (Nigeria, Ghana and Benin). An additional 688-nt deletion was observed between positions 8,557 and 9,245, resulting in in-frame fusion of *MGF 110–3L* and *MGF 110–4L*, as shown in Fig. 3b(ii). Phylogenetic analysis reveals a common ancestral deletion of 688 and 6,535 in genotype II from West Africa (Fig. 3b, i). Further comparison between the genotype II strains from Nigeria and Georgia 2007/1 revealed unique SNPs. Nigeria-LA74 has 60 SNPs, and Nigeria-613 has 68 SNPs, with a single-nucleotide deletion of ‘A’ on MGF 505–4R at position 1,465. However, to obtain a functionally relevant annotation, Nigeria-RV502 was used as a reference because of its high genetic similarity. Note: due to the high error rate of ONT, Nigeria-DDS922 was excluded from the SNP analysis. All the genomic changes from different years of outbreaks for genotypes I and II in Nigeria showed evidence that the ASFV is evolving, and there is a need for incorporation of relevant changes for a live attenuated vaccine that will be effective in controlling the disease.

### Purifying and diversifying selection pressure

The evolutionary analysis of potential site-specific selection pressure, as measured by the ratio of non-synonymous (dN) to synonymous (dS) substitutions. Gene *DP71L* showed evidence of diversifying selection using FUBAR analysis with a posterior probability of 0.9, while with the default threshold of MEME with *P*-value 0.1, six sites in five genes, *Q706L*, *MGF 505–2R*, *P1192R*, *B407L* and *MGF 360–8L*, were detected to be under diversifying positive selection; after re-evaluating the *P*-value 0.05, only three sites on three genes, *P1192R*, *B407L* and *MGF 360–8L*, showed evidence of diversifying positive selection. No evidence of selection was detected using SLAC analysis (Table S4).

## Discussion

To the best of our knowledge, this is the first work that simultaneously reports hybrid whole genomes of genotypes I and II from Africa and also the first report to generate a high-quality hybrid assembly of genotype I from Nigeria since it was first reported in 1997 [58]. Evolutionary analysis of genotypes I and II provides insights into ASF movements within West Africa and across the continent, revealing the ancestral origin of the circulating strains in Nigeria and Cameroon. The report also highlights recent genomic changes of the virus from different outbreaks over time in Nigeria: Benin 97/1 to Nigeria-VPD805 with 36 SNPs, Benin 97/1 to Nigeria-DDS943 with 39 SNPs, Nigeria-VPD805 to Nigeria-DDS943 with 13 SNPs, Nigeria-RV502 to Nigeria-LA74 with 4 SNPs, Nigeria-RV502 to Nigeria-613 with 16 SNPs and Nigeria-LA74 to Nigeria-613 with 14 SNPs. And also, selection analysis highlights *P1192R*, *B407L* and *MGF 360–8L* as candidate genes that may be under selection pressure.

The genetic characterization of ASFV on the continent of Africa, based on the assembled genomes reported in this study, makes a substantial contribution to the currently very limited whole-genome data available for ASFV from Africa. The diversity and evolution of ASFV in West Africa are now better understood following this report. Our data also provide insights into the choice of isolates as a basis for the development of vaccines. The evolutionary analysis of the two genotypes offers a valuable insight into continent-wide transmission and the possible introduction routes of ASFV into Nigeria. TMCA of genotype I have linked the West African strains to strain E75 (Spain) from Europe several decades ago, which is in line with the historic linkage between Europe and West Africa [59]. This has reflected the transcontinental animal movement between Europe and Africa [60]. The TMCA of genotype II divergence between African strains and the Eurasian is estimated to be 23.8 years ago. However, the recent emergence of genotype II in Nigeria in 2020 shared the same ancestral origin as that of the 2022 report from Ghana ~8 years ago, as shown in Fig. 2b. The gap between the divergence of the West African genotype II from the Southeast African strains and the time of the outbreak reflects the period of undetected circulation of the virus on the continent, possibly in its ancestral host, the warthog or in the different epidemiological circumstances prevalent in West Africa. The more rapid evolution of genotype II observed in this report, compared to the previous studies from Southeast Africa, including Tanzania [61], may well reflect the adaptation of ASFV to West African pig populations.

Comparative genomic analysis of genotype I using reference Benin 97 revealed a progressive pattern of genomic changes; a deletion of 38 nt from 1,077 to 1,115 on the *B475L* gene was identified only in strain Nigeria-VPD805 and Nigeria-DDS943 (2021/2023 outbreaks), which is absent in those of the 2016 outbreak. Given the function of the *B475L* gene inhibiting interferon (IFN-I) by inhibiting signal transduction protein and activator of transcription (STAT1 and STAT2), heterodimerization and nuclear translocation, respectively [62]. Despite the deletions, the virus remains virulent, suggesting that the genetic changes did not compromise the virus’s ability to evade the host’s immune and inflammatory responses. However, genes like *MGF 360–9L* and *MGF 505–7R* have also been reported to have a similar function [63,64]. Comparison between the early report of genotype II and the newly assembled sequences provides evidence that the virus is evolving; new observed SNPs are present in the Nigeria-613 isolate from the 2024 outbreak. Further comparison of genotype II (Nigeria-LA74 and Nigeria-613) with a standard reference, Georgia 2007, revealed a 6,535-nt deletion that resulted in the loss of 14 genes, and a 688-nt deletion led to the in-frame fusion of *MGF 110–3L* and *MGF 110–4L*. This variation was observed in Nigeria-RV502 and Ghana2022-35 strains [25,65]. Many of these deleted genes have no clearly identified function in contributing to the virus’s virulence, except for *MGF 360–4L*, a gene reported to have the function of ‘inhibiting interferon signalling by recruiting mitochondrial selective autophagy receptor SQSTM1, degrading MDA5 and antagonizing innate immune responses’ [66]. The *MGF-110–9L* gene has been shown to function in reducing virulence after deletion [67]. Deletions in the ASFV genotype II were also observed in samples from Italy and Estonia-2014 strains [68,69]. However, these deletions are not identical to the West African genotype strains. The role of genetic variation, especially genotype II, needs to be ascertained. Despite the large deletion observed in *MGF* in the genotype II strain, field observation showed that the virus is still characterized by a high mortality rate. Control experimental infection and virulence studies are needed to understand the function and the implications of these deletions. We also identify gene *P1192R*, *B407L* and *MGF 360–8L* to be under diversifying selection. Gene *P1192R* has been reported earlier with a role involving viral replication [70], while *B407* and *MGF 360–8L* have no identified function. Although the changes in genotype II, after transmission to Europe and East Asia, are not identical to those in Nigeria, it is interesting to note that multiple genotype IX genomes from the endemic region of East Africa (East Uganda to central Kenya) exhibit relatively little difference [61].

Although the study sheds important light on different genotypes of ASFV that are circulating in Nigeria and the evolution of the virus, our conclusions are limited by the limited number of available genomic sequences. We acknowledge the reports of known error sequences previously published in this report (MN336500, MN394630, MN648177 and MN641876). Subsequent changes to these records have been noted with the newly sequenced genome PQ035960; this did not affect the tree topology, the placement of the newly sequenced genome and the validity of our conclusion as shown in Fig. S1 [71]. More understanding of genomic diversity, selection pressures and the evolution trajectory of the virus in the region will be aided by additional sequence and metadata from Nigeria and other West African nations (Burkina Faso, Togo and Côte d’Ivoire). In conclusion, the study presents a high-quality assembled genome of Nigerian ASFV genotypes I and II. Using phylogenetic analysis, genotype I ancestral lineage was found to be Cameroon, while genotype II was found to be Mauritius (presumably, ultimately originating from Madagascar or South East Africa). Novel genomic changes were also reported in both genotypes I and II, with significant implications for future surveillance and ASFV control.

## Supplementary material

10.1099/mgen.0.001636 Uncited Fig. S1.

10.1099/mgen.0.001636 Uncited Table S1.

10.1099/mgen.0.001636 Uncited Table S2.

10.1099/mgen.0.001636 Uncited Table S3.

10.1099/mgen.0.001636 Uncited Table S4.

## Abbreviations

ASF: African swine fever
ASFV: African swine fever virus
BIC: Bayesian information criterion
CCR: central conserved region
HPD: Highest Posterior Density interval
LVR: left variable region
MCMC: Markov Chain Monte Carlo
MEME: mixed effects model of evolution
NGS: next-generation sequencing
NVRI: National Veterinary Research Institute
ONT: Oxford Nanopore Technology
RVR: right variable region
TMCA: time of the most common ancestry
