# Supplementary material for: Genome sequencing reveals variation of African swine fever virus in Nigerian outbreaks and identification of two major West African viral lineages
Source: Microb Genom. 2026 Feb 10;12(2):001636. doi: 10.1099/mgen.0.001636 (PMC13293334; doi:10.1099/mgen.0.001636)

Supplementary Figure 1:

Maximum likelihood phylogenetic tree of the five (5) new Nigerian ASFV genomes (highlighted in blue with an asterisk) together with 344 previously published ASFV whole genomes. Reported known error sequences were excluded: MN336500, MN394630, MN648177 and MN641876. A re-sequenced genome, **PQ035960** (In bold) was included for phylogenetic tree comparison. the phylogenetic tree is rooted at the midpoint. All branches shown are supported by 70% or higher bootstrap values. Colors represent genotypes of ASFV based on the p72 gene. Highlighted in light yellow are reported recombinant genomes. Seven recombinant genomes between genotypes I and II show as a distinct clade.

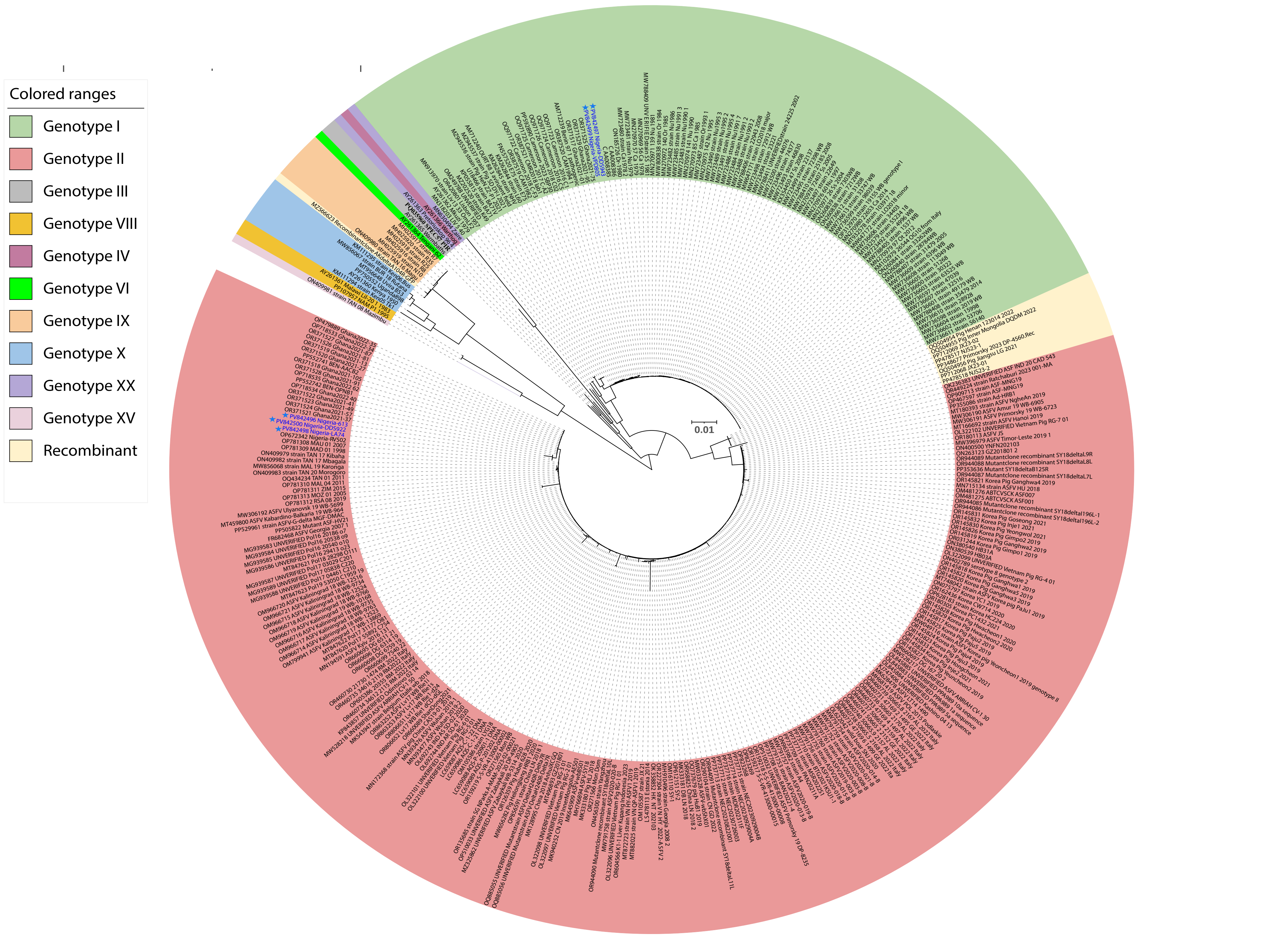

Supplement: Uncited Fig. S1. [file mgen-12-01636-s001.pdf]
