# Supplementary material for: Genome sequencing reveals variation of African swine fever virus in Nigerian outbreaks and identification of two major West African viral lineages
Source: Microb Genom. 2026 Feb 10;12(2):001636. doi: 10.1099/mgen.0.001636 (PMC13293334; doi:10.1099/mgen.0.001636)
Supplement: Uncited Table S4. [file mgen-12-01636-s005.pdf]

Supplementary Table 4 Selection pressure of genes with mutation. The threshold of significance (P < 0.05) was used for MEME and SLAC, and a posterior probability of 0.9 for FUBAR. Evidence of diversifying selection are highlighted in red color.

**Proteins**	**SLAC**		**FUBAR**		**MEME**		**Position**	**Remark**	**Biological Relevance**
	dN-dS	P{dN/dS} <1	B-α	prob[α<β]	β+	P-Value			
DP71L	14.0	1.00	20.995	**0.948**	59.98	0.145	21	Neutral	Regulate ER stress-mediated apoptosis
P1192R	23.0	1.00	9.151	0.738	7,638.656	**0.029**	1096	Diversifying	Involved in viral replication
B407L	17.5	1.00	11.270	0.779	2,621.88	**0.039**	103	Diversifying	Unknown function
MGF 505-2R	43.5	1.00	16.419	0.828	4,539.448	0.054	308	Neutral	Immune invasion
MGF 360-8L	16.4	1.00	15.968	0.834	2,913.031	**0.046**	35	Diversifying	Unknown function
B438L	58.1	1.00	17.262	0.841	1,874.919	0.163	203	Neutral	Viral assembly and release
E301R	47.4	1.00	18.571	0.836	1,609.997	0.174	276	Neutral	Viral genome replication
I267L	18.4	1.00	11.092	0.791	1,472.049	0.102	196	Neutral	Inhibits RNA polymerase III-RIG-I-mediated innate immunity
